# Long Noncoding RNA RP11-115N4.1 Promotes Inflammatory Responses by Interacting With HNRNPH3 and Enhancing the Transcription of HSP70 in Unexplained Recurrent Spontaneous Abortion

**DOI:** 10.3389/fimmu.2021.717785

**Published:** 2021-08-13

**Authors:** Meilan Liu, Xiaoyue Sun, Liqiong Zhu, Menglan Zhu, Kewen Deng, Xiaolu Nie, Hanjie Mo, Tao Du, Bingqian Huang, Lihao Hu, Liuhong Liang, Dongyan Wang, Yinger Luo, Jinling Yi, Jianping Zhang, Xingming Zhong, Chunwei Cao, Hui Chen

**Affiliations:** ^1^Department of Obstetrics and Gynecology, Sun Yat-Sen Memorial Hospital, Sun Yat-Sen University, Guangzhou, China; ^2^Guangdong Provincial Key Laboratory of Malignant Tumor Epigenetics and Gene Regulation, Guangdong-Hong Kong Joint Laboratory for RNA Medicine, Sun Yat-Sen Memorial Hospital, Sun Yat-Sen University, Guangzhou, China; ^3^Medical Research Center, Sun Yat-Sen Memorial Hospital, Sun Yat-Sen University, Guangzhou, China; ^4^Center for Reproductive Genetics and Reproductive Medicine, Sun Yat-Sen Memorial Hospital, Sun Yat-Sen University, Guangzhou, China; ^5^Department of Gynecology, The Fifth Affiliated Hospital of Xinjiang Medical University, Urumqi, China; ^6^Key Laboratory of Male Reproduction and Genetics of National Health Council, Family Planning Research Institute of Guangdong Province, Guangzhou, China; ^7^Department of Public Health and Preventive Medicine, School of Medicine, Jinan University, Guangzhou, China; ^8^Department of Genetics and Cell Biology, Zhongshan School of Medicine, Sun Yat-Sen University, Guangzhou, China

**Keywords:** unexplained recurrent spontaneous abortion, long noncoding RNA, RP11-115N4.1, HNRNPH3, HSP70, inflammatory response

## Abstract

**Background:**

Unexplained recurrent spontaneous abortion (URSA) is a common pregnancy complication and the etiology is unknown. URSA-associated lncRNAs are expected to be potential biomarkers for diagnosis, and might be related to the disease pathogenesis.

**Objective:**

To investigate differential lncRNAs in peripheral blood of non-pregnant URSA patients and matched healthy control women and to explore the possible mechanism of differential lncRNAs leading to URSA.

**Methods:**

We profiled lncRNAs expression in peripheral blood from 5 non-pregnant URSA patients and 5 matched healthy control women by lncRNA microarray analysis. Functions of URSA-associated lncRNAs were further investigated *in vitro*.

**Results:**

RP11-115N4.1 was identified as the most differentially expressed lncRNA which was highly upregulated in peripheral blood of non-pregnant URSA patients (*P* = 3.63E-07, Fold change = 2.96), and this dysregulation was further validated in approximately 26.67% additional patients (4/15). RP11-115N4.1 expression was detected in both lymphocytes and monocytes of human peripheral blood, and *in vitro* overexpression of RP11-115N4.1 decreased cell proliferation in K562 cells significantly. Furthermore, heat-shock HSP70 genes (*HSPA1A* and *HSPA1B*) were found to be significantly upregulated upon RP11-115N4.1 overexpression by transcriptome analysis (*HSPA1A* (*P* = 4.39E-08, Fold change = 4.17), *HSPA1B* (*P* = 2.26E-06, Fold change = 2.99)). RNA pull down and RNA immunoprecipitation assay (RIP) analysis demonstrated that RP11-115N4.1 bound to HNRNPH3 protein directly, which in turn activate heat-shock proteins (HSP70) analyzed by protein-protein interaction and *HNRNPH3* knockdown assays. Most importantly, the high expression of HSP70 was also verified in the serum of URSA patients and the supernatant of K562 cells with RP11-115N4.1 activation, and HSP70 in supernatant can exacerbate inflammatory responses in monocytes by inducing IL-6, IL-1β, and TNF-α and inhibit the migration of trophoblast cells, which might associate with URSA.

**Conclusion:**

Our results demonstrated that the activation of RP11-115N4.1 can significantly increase the protein level of HSP70 *via* binding to HNRNPH3, which may modulate the immune responses and related to URSA. Moreover, RP11-115N4.1 may be a novel etiological biomarker and a new therapeutic target for URSA.

## Introduction

Recurrent spontaneous abortion (RSA), defined as the loss of ≥ 3 consecutive pregnancies before 24th gestational week, affects 1%-5% women during their reproductive age (1). Couples who experience RSA are under great mental stress. The incidence of RSA is on the rise around the world, especially due to the increase in the age of pregnant women and the deterioration of environment (2). Moreover, epidemiological studies have found that pregnancies after RSA increased the risk of pregnancy complications, such as preeclampsia, fetal growth restriction, preterm birth and stillbirth (2, 3). The etiology of RSA is complicated, and some factors are known to lead to RSA, including abnormal chromosome karyotype of parents or embryos, abnormal uterus anatomy, endocrine and metabolic abnormalities, infectious diseases, antiphospholipid syndrome and environmental factors (3, 4). However, there are still 50%-60% of cases who cannot be explained by above causes, and the cellular and molecular pathways leading to RSA are not be fully understood (1, 3–5). Current studies suggest that URSA is related to immune abnormalities (1, 5–8).

Long noncoding RNA (lncRNA) is a class of noncoding RNAs that are longer than 200 nt and is transcribed from exons, introns and gene deserts (9). LncRNAs have been revealed to play important roles in various cellular functions, including affecting the pluripotency and differentiation of embryonic stem cells, regulating cell apoptosis and cell cycle, and performing important functions in the origin, growth, evolution, selection and other aspects of human life (9–11). Recent several researches have found that lncRNAs may be related to RSA (12, 13). However, there is only one study investigating relation of lncRNA with unexplained recurrent pregnancy loss (URPL) (14). The results showed that lnc-SLC4A1-1 was highly expressed in villi of URPL patients and facilitated trophoblast cell migration and apoptosis (14). However, whether lncRNAs in peripheral blood immunocytes of URSA patients in non-pregnant state can serve as biomarker of URSA or be related to the disease onset has not been studied extensively, and needs further investigation.

In this study, we investigated the differential expressed lncRNAs in peripheral blood between non-pregnant URSA patients and non-pregnant healthy women. The possible regulation mechanism of candidate lncRNAs involved in URSA are further explored *in vitro*. These findings not only deepen our understanding of the mechanism of URSA but also provide novel targets for predictive diagnosis and intervention therapy of URSA.

## Materials and Methods

### Study Population

Non-pregnant URSA patients and healthy control women were enrolled from Sun Yat-Sen Memorial Hospital from Jan 2017 to Sep 2017 and from Dec 2020 to May 2021. URSA was defined as the loss of ≥ 3 consecutive pregnancies before 24th gestational week (1). In accordance with the hospital protocol for women with RSA, all women with RSA underwent a systematic examination for all causes of RSA during their first visit (15). Women were excluded if 1) they had parental or embryonic chromosomal abnormalities, any infections, uterine abnormalities, endocrine diseases, and anti-phospholipid antibody syndrome responsible for previous pregnancy losses; 2) they had autoimmune diseases, especially rheumatic immune diseases; 3) their RH blood was negative; 4) semen results of patient’s husband were abnormal. The inclusion criteria for control subjects included ≥ 1 successful pregnancy and no previous miscarriage. The exclusion criteria for all subjects were as follows: they were pregnant or had bad lifestyle habits, such as drinking, smoking, or had medical diseases, or had been vaccinated before enrollment and had taken drugs within 90 days that may affect immune function.

The clinical features, immune and biochemical markers levels were recorded. Regular menstruation was required as follows: 1) menstrual cycle ranges from 21 to 35 days; 2) menstruation period lasts between 3 and 7 days; 3) no irregular menstrual bleeding or dysmenorrhea. Assisted reproduction and regular menstruation period were recorded as yes or no.

The study was approved by the Ethics Committee of Sun Yat-Sen Memorial Hospital (No. SYSEC-KY-KS-2019-379). After fully understanding the study, all subjects signed informed consent.

### Immune and Biochemical Marker Measurements

Abbreviations are used to describe the immune and biochemical markers, and their normal reference values, full name and unit are shown in **Supplementary Table 1**. Mixed lymphocyte reaction-blocking factors was tested by ELISA and the original matching kit (Sysmex XN9000, Japan). The lymphocyte subgroup was performed by flow cytometry (BD FACS Canto II, USA). Anti-cardiolipin antibody- immunoglobulin G/M/A (ACA-IgG/M/A), anti-β2-Glycoprotein 1 antibody- immunoglobulin G/M/A (Anti-β2GP1-Ab-IgG/M/A), anti-Phosphatidylserine/Prothrombin antibody-immunoglobulin G/M (aPS/PT-IgG/M) were measured by chemiluminescence and the original matching kit (INOVA Co., USA). Homocysteine (Hcy) was performed by enzyme circulation method and the original matching kit (Meikang Biological Technology Co., Ltd, Ningbo, China). Platelet aggregation function (adenosine diphosphate, ADP) was tested using photoelectric turbidimetry (PrecilInstrument Co., Ltd, Beijing, China).

### LncRNA Microarray Analysis

LncRNA microarray analysis was performed in 5 URSA patients and 5 healthy controls using Arraystar Human LncRNA Microarray V4.0 (Agilent Technology, including 40,173 lncRNA and 20,730 mRNA targets) by KangChen Bio-Tech (Shanghai, China). The hybridized arrays were scanned using the Agilent DNA Microarray Scanner, and the acquired array images were then analyzed *via* Agilent Feature Extraction software (version 11.0.1.1). The normalization of lncRNA expression data was further performed using the GeneSpring GX v12.1 software package (Agilent Technologies).

### RNA Sequencing and Analysis

Total RNA isolation was performed using the Trizol reagent (Invitrogen, USA), and the quality and purity of total RNA was tested using Nano Drop ND-1000 spectrophotometer (Nano Drop, DE, USA) and Agilent 2100 Bioanalyzer (Agilent, Santa Clara, CA). Paired-end (PE) libraries were prepared following the illumina paired-end library preparation protocol (Illumina, San Diego, CA), and were then sequenced on an Illumina NovaSeq sequencing platform to generate 2 × 150 paired-end reads.

The RNA-seq reads were first processed using the Trimmomatic program (16) to detect adapter contamination and remove sequencing reads with low quality bases with the following command ‘adapters.fa:2:30:10 LEADING:3 TRAILING:3 SLIDINGWINDOW:4:15 MINLEN:50’. The high-quality reads were then aligned to human GRCh37 reference genome using TopHat software (17) with default parameters. Next, the number of uniquely mapped reads assigned to each gene in human genome was counted using featureCounts tool, and edgeR software (18), using the read counts as input, was then applied to identify differentially expressed genes using a threshold value of FDR values < 0.05 and absolute fold change ≥ 2 (log2FC ≥ 1). Gene ontology enrichment analysis was performed on the significantly upregulated and downregulated genes using Shiny webtool (http://bioinformatics.sdstate.edu/go/) (19). The GO terms with P values (enrichment score) < 0.05, were regarded as “statistically significant”. The protein-protein interaction network was constructed using STRING webtool (http://string=db/org).

### Cell Culture

K562 as chronic myelogenous leukemia cell line was purchased from Cell cook Biotech. The K562 Cells were cultured in Iscove’s Modified Dulbecco Medium (IMDM) (HyClone, Logan, UT) containing 10% FBS, 100 IU/mL penicillin and 100 µg/mL streptomycin (Invitrogen, CA, USA) at 37°C in a humidified 5% CO_2_ incubator.

### Gene Overexpression

The K562 cells with RP11-115N4.1 overexpression was constructed as follows: RP11-115N4.1 cDNA was first cloned into the pCDH-CMV-MCS-EF1-GFP vector (the empty vector was used as a negative control). Then, 10 μg expression vector, 7.5 μg pCMV-VSVG and 7.5 μg psPAX2 were co-transfected into 293T packaging cells by LentiFit (Hanbio Biotechnology), and viral supernatant was harvested at 48 h or 72 h after transfection. Finally, the K562 cells were infected with the viruses at 37°C for 24 h, and the positive cells were selected by incubating with 1 µg/mL puromycin.

### RNA Isolation and Quantitative Real-Time PCR

Total RNA was extracted from K562 cells using Trizol (Invitrogen, Carlsbad, USA). Reverse transcription of RNA into cDNA was performed using the ReverTra Ace qPCR RT kit (Toyobo, Osaka, Japan). Real-time reverse transcription-PCR (RT-PCR) was then carried out by Bio-Rad Real-Time PCR Detection system (CFX Connect, USA). *GAPDH* was used as an endogenous control. The RT-PCR primers were listed in **Supplementary Table 2**.

### RNA Pull-Down Assay

The synthetic RP11-115N4.1 plasmid was used as a template to synthesize the biotinylated RP11-115N4.1 and antisense transcript using the T7 *in vitro* transcription kit (Thermo Fisher Scientific, US). A streamlined procedure was provided by Pierce magnetic RNA-Protein Pull-down Kit (Thermo Fisher Scientific, MA, USA). First, T4 RNA ligase attaches a single desthiobiotinylated cytidine bisphosphate to the 3’ end of the RNA strand at 16°C overnight. Second, incubating the labeled RNA with streptavidin magnetic beads at room temperature for 30 min with rotation to bind them. Third, incubating the RNA-bound streptavidin magnetic beads with cell lysates at 4°C for 1 h with rotation. Finally, identification of the pull-down proteins by mass spectrometry (Thermo Fisher Nano1200-Fusion orbitrap) and silver stain. The RP11-115N4.1-bound protein HNRNPH3 was then tested by Western blot.

### RNA Immunoprecipitation Assay (RIP)

RIP assays were performed to determine whether HNRNPH3 was interacted with RP11-115N4.1 using the EZMagna RIP Kit (Millipore, Billerica, MA, USA). A mouse antibody against the HNRNPH3 (Santa Cruz) was used for the RIP assays, and qRT-PCR analysis was further performed to measure the expression level of RP11-115N4.1. Normal mouse IgG (Millipore, Billerica, MA, USA) was used as a negative control. The primers used to detect RP11-115N4.1 and *GAPDH* were listed in **Supplementary Table 2**.

### Western Blotting Assay

The K562 cell proteins were lysed with radio-immunoprecipitation assay buffer and the protein concentrations were determined by BCA assay (Beyotime Institute of Biotechnology). Equal amounts of protein were separated by the SDS polyacrylamide gel. The proteins were transferred to polyvinylidenedifluoride membranes (Millipore) and blocked with 5% skim milk. The membranes were incubated overnight with anti-human HSPA1A (Proteintech), anti-human HNRNPH3 (Santa Cruz), or anti-human β-Actin (Affinity) antibodies. After washing, the membranes were incubated with the appropriate secondary antibody (Santa Cruz) for visualizing proteins by chemiluminescence reagent. The intensity of the Western blot bands were quantified by Image J software.

### Enzyme-Linked Immunosorbent Assay (ELISA)

The HSP70 (HSPA1A), IL-6, TNF-α and IL-1β in serum or culture supernatant were analyzed using the ELISA kits (Cusabio Technology llc, Houston, TX, USA). Samples or standards were first added to each well and incubated for 2 hours at 37°C. Then, after removing the supernatant, the biotin-conjugated antibody was added to each well and incubated for 1 hour. After washing, horseradish peroxidase (HRP) was added to each well, and the substrate solution was added to generate a color. The stop solution was added and the color intensity was measured at 450 nm.

### Statistical Analysis

SPSS were used in this study (version 20.0). Continuous data were described using mean ± standard deviation, or median with range (minimum, maximum), and were analyzed with Student *t*-test, Wilcoxon test, as appropriate. Categorical data were reported as numbers and percentages, and were analyzed with the χ^2^ test or Fisher’s exact test as appropriate. *P* < 0.05 was considered to be statistical significance.

## Results

### RP11-115N4.1 Expression Significantly Increased in PBMC of URSA

To identify the lncRNAs that are potentially involved in URSA, we profiled lncRNA expression from peripheral blood mononuclear cell (PBMC) of URSA patients and normal controls (5 *vs.* 5). The clinical features of 10 participants were shown in **Table 1**. The URSA and control groups were well matched, and no statistical differences were found in clinical features between URSA patients and normal control women other than the number of gravidities, live births and spontaneous abortions.

**Table 1:** Clinical features of URSA patients and normal control women with lncRNA microarray analysis (mean ± SD or number/percentage or median with range (minimum, maximum)).

Variables	URSA (*N*=5)	Control (*N*=5)	*Z*	*P*-value
**Age (year)**	30 (23, 30)	32 (28, 33)	-0.843	0.339
**Height (cm)**	157.10 ± 4.01	160.60 ± 4.98	~	0.256
**BMI (kg/m^2^)**	20.32 ± 3.13	22.24 ± 3.28		0.372
**Local**				
Urban	2 (40%)	5 (100%)	~	0.167
Rural	3 (60%)	0 (0%)		
**Regular menstruation period**				
Yes	2 (40%)	3 (60%)	~	1.000
No	3 (60%)	2 (40%)		
**Previous assistant reproduction**				
Yes	1 (20%)	0 (0%)	~	1.000
No	4 (80%)	5 (100%)		
**Number of gravidities**	**4.00 ± 0.71**	**1.00 ± 0.00**	****	**0.000**
**Number of live births**	**0 (0, 1)**	**1 (1, 1)**	**-2.449**	**0.014**
**Number of spontaneous abortions**	**3 (3, 5)**	**0 (0, 0)**	**-2.887**	**0.004**

SD, standard deviation; URSA, unexplained recurrent spontaneous abortion. The bold value represents p < 0.05.

**Figure 1:**
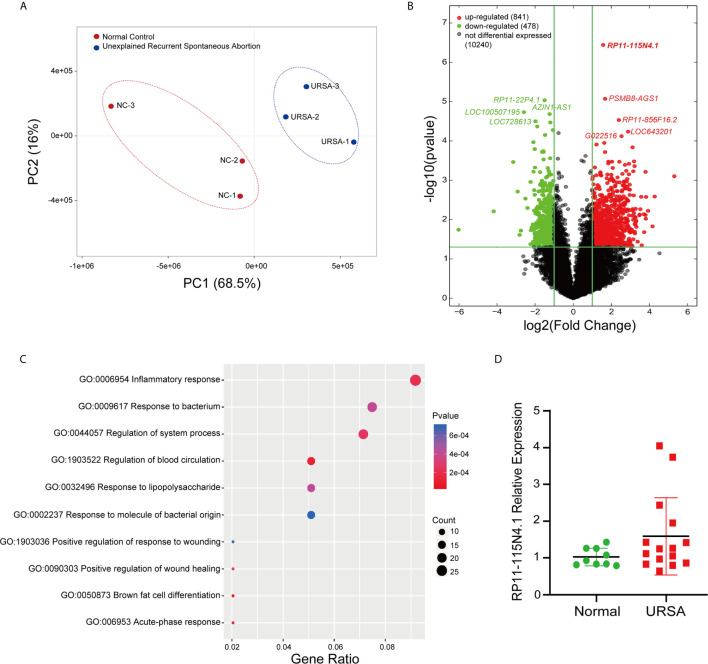
RP11-115N4.1 Expression Significantly Increased in PBMC of URSA. **(A)** Cluster map of URSA samples and normal controls (3 *vs.* 3). **(B)** Volcano plot described that the differentially expressed lncRNAs in URSA (Fold change ≥ 2, *P* < 0.05). **(C)** GO analysis indicated that inflammatory response pathway was significantly enriched in URSA. **(D)** Quantitative RT-PCR analysis of RP11-115N4.1 level in URSA blood samples (URSA, *N* = 15) and normal woman blood samples (Normal, *N* = 9). *GAPDH* was used as the internal control.

The PCA analysis indicated the heterogeneity of lncRNA expression profiles in the study population (**Supplementary Figure 1**), and thus 3 URSA and 3 normal control women which grouped into separate clusters were selected for differential expression analysis (**Figure 1A**). The differentially expressed lncRNAs were displayed by volcano plots (**Figure 1B**), showing that 841 lncRNAs were significantly upregulated and 478 lncRNAs were significantly downregulated in URSA (Fold change ≥ 2, FDR < 0.05). Among them, lncRNA RP11-115N4.1 showed the most upregulated expression in URSA (*P* = 3.63E-07, Fold change = 2.96), **Figure 1B**). Furthermore, GO analysis using the significantly deregulated mRNA indicated that the top enriched upregulated pathway was inflammatory response (**Figure 1C**). To estimate the percentage of URSA patients with lncRNA RP11-115N4.1 high expression, another 15 URSA samples and 9 normal controls were collected to detect the expression of RP11-115N4.1 in PBMC. The results showed that among the 15 URSA samples, the expression of RP11-115N4.1 in 4 samples was higher than that in the normal group, but the difference was not significant (**Figure 1D**). Therefore, we focused on RP11-115N4.1 for further study.

### Differences in Clinical Features, Immune and Biochemical Levels of URSA Patients With High and Normal Expression of RP11-115N4.1

Based on the expression of RP11-115N4.1, 20 patients with URSA were divided into group 1 with high expression of RP11-115N4.1 (*N* = 7) and group 2 with normal expression of RP11-115N4.1 (*N* = 13). As shown in **Supplementary Table 3**, there were no statistical difference among the clinical features, immune and biochemical levels between group 1 and group 2. Interestingly, 3 of 13 in group 2 had previous late miscarriage, while none was found in group 1.

### RP11-115N4.1 Overexpression Significantly Decreased K562 Cell Proliferation

**Figure 2:**
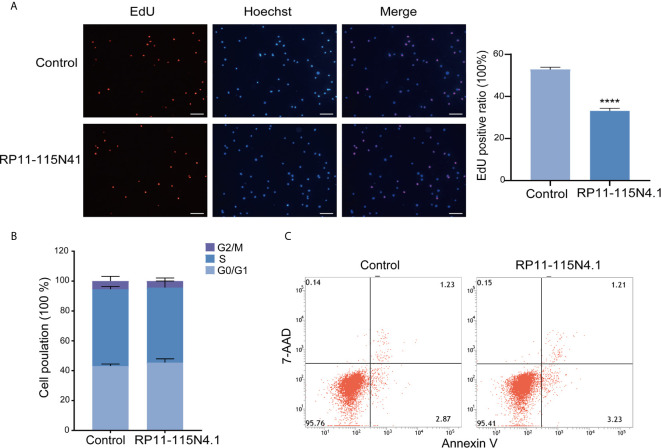
RP11-115N4.1 overexpression significantly decreased K562 cell proliferation. **(A)** EdU assay for cell proliferation in K562 cells with RP11-115N4.1 overexpression. The percentage of EdU positive cells was detected by flow cytometry. The picture scale was 100 *μ*m. **(B)** Cell cycle assay of K562 cells with RP11-115N4.1 overexpression. **(C)** Cell apoptosis analysis in K562 cells with RP11-115N4.1 overexpression. ****P < 0.0001. Each experiment was repeated three times and results are means ± SD.

The expression of RP11-115N4.1 was analyzed in both lymphocytes and monocytes in human peripheral blood, and the results showed that both were expressed, and the difference was not significant (**Supplementary Figure 2**). Here, the human lymphoblast cell line K562 was used to study the RP11-115N4.1’s function. To determine whether RP11-115N4.1 have regulatory effects on K562 cell function, we overexpressed RP11-115N4.1 in K562. After transfection, the expression level of RP11-115N4.1 was significantly higher than that in controls (**Supplementary Figure 3**). Cell proliferation was significantly decreased in K562 cells after overexpression of RP11-115N4.1 (**Figure 2A**). While, the RP11-115N4.1 overexpression had no effects on cell cycle and cell apoptosis (**Figures 2B, C**).

### RP11-115N4.1 Promoted HSP70 Transcription

**Figure 3:**
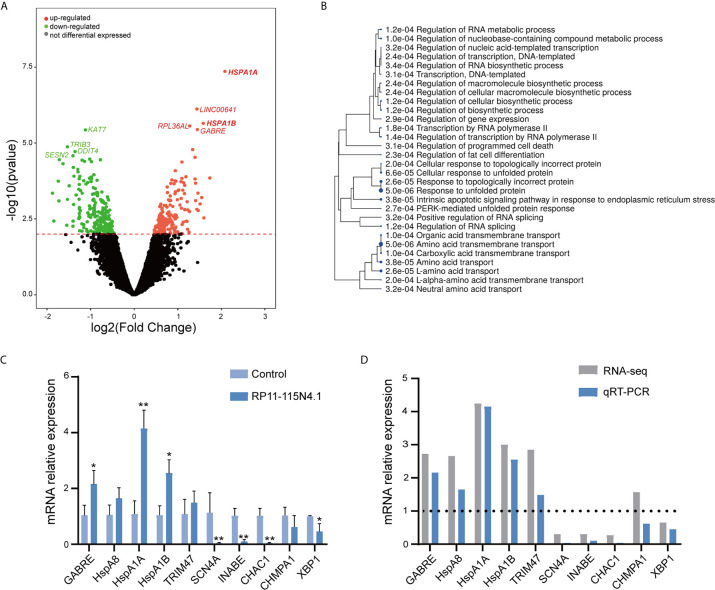
RP11-115N4.1 promoted HSP70 transcription. **(A)** Volcano plot described that the differentially expressed mRNAs in K562 cells with RP11-115N4.1 overexpression. **(B)** GO analysis data described that the differentially expressed mRNAs in K562 cells with RP11-115N4.1 overexpression. **(C, D)** Validation of differentially expressed genes by qRT-PCR. *P < 0.05, **P < 0.01. Each experiment was repeated three times and results are means ± SD.

To further evaluate the function of RP11-115N4.1 in K562 cells, we performed RNA-seq in K562 cells overexpressing RP11-115N4.1 (**Figure 3A**). Most interesting, the RNA-seq suggested that RP11-115N4.1 significantly upregulated the expression of HSP70 related genes (*HSPA1A* (*P* = 4.39E-08, Fold change = 4.17), *HSPA1B* (*P* = 2.26E-06, Fold change = 2.99)), and response to unfold protein pathway was strongly enriched in the GO analysis (**Figure 3B**). In addition, we used qRT-PCR to validate the differentially expressed genes identified in RNA-seq (**Figures 3C, D**), and the results indicated that qPCR results were consistent with RNA-seq results. Compared with the control, we found that RP11-115N4.1 significantly promoted the expression levels of *HSPA1A*, *HSPA1B* and *GABRE* in K562 cells.

### RP11-115N4.1 Promoted HSP70 Expression by Interacting With HNRNPH3

**Figure 4:**
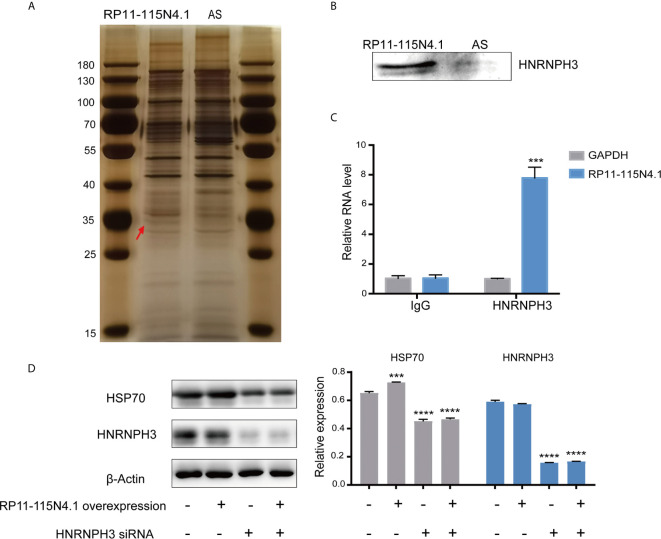
RP11-115N4.1 promoted HSP70 expression by interacting with HNRNPH3. **(A, B)** RNA-pull-down assay to identify RP11-115N4.1 binding protein in K562 cells. The eluted proteins were separated by SDS-PAGE and subjected to silver staining. Antisense RNA to RP11-115N4.1 (AS) was used as a negative control. The red arrow indicates the band representing the RP11-115N4.1-specific binding protein identified by mass spectrometry as HNRNPH3. Western blot analysis confirmed that RP11-115N4.1 interacts with HNRNPH3. **(C)** RIP assay was performed using normal mouse IgG or the anti-HNRNPH3 antibody. *GAPDH* was used as the negative control. **(D)** Western blot of HSP70 and HNRNPH3 in K562 cells under different condition. *β*-Actin was used as an internal control. ***P < 0.001, ****P < 0.0001. Each experiment was repeated three times and results are means ± SD.

It is known that lncRNA generally perform their functions by coordinating with protein partners (20). To investigate the mechanism by which RP11-115N4.1 activated HSP70, we used RNA pull-down assays to identify proteins that directly bind to RP11-115N4.1. Mass spectrometry analysis of the eluted protein showed that RP11-115N4.1 RNA specifically associated with heterogeneous ribonucleoprotein H3 (HNRNPH3) (**Figures 4A, B** and **Supplementary Table 4**). A RIP assay was subsequently performed in K562 cells using the anti-HNRNPH3 antibody, and the results confirmed that HNRNPH3 bound to RP11-115N4.1 (**Figure 4C**). Furthermore, the protein-protein interaction network showed that there was an indirect connection between HNRNPH3 and HSP70 (**Supplementary Figure 4**). Hence, the regulatory relationship between HNRNPH3 and HSP70 was further investigated (**Figure 4D** and **Supplementary Figures 5A, B** **)**, and the results showed that knocking down HNRNPH3 resulted in the significant reduction of HSP70 mRNA and protein expression (*P*<0.01).

### The Supernatant of K562 Cells Overexpressed RP11-115N4.1 Induced the Inflammatory Response of Monocytes and Inhibited the Migration of Trophoblast Cells

**Figure 5:**
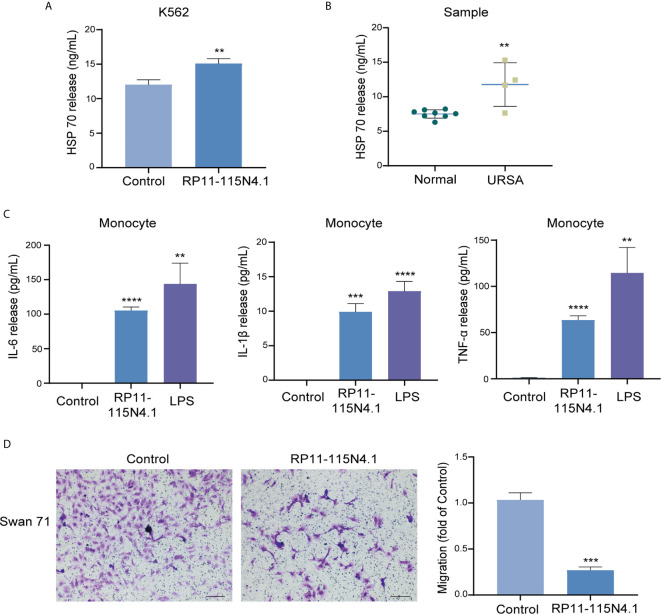
The supernatant of K562 cells overexpressed RP11-115N4.1 induced the inflammatory response of monocytes and inhibited the migration of trophoblast cells. **(A)** The level of HSP70 was determined by ELISA in K562 cell treated with RP11-115N4.1. **(B)** The level of HSP70 was determined by ELISA in the serum of URSA samples with high RP11-115N4.1 expression. **(C)** The levels of IL-6, IL-1β and TNF-α were determined by ELISA in human monocyte under different condition. LPS was used as a positive control (100 μg/mL). **(D)** Transwell assay in Swan 71 cell under different condition. The picture scale was 100 *μ*m. **P < 0.01, ***P < 0.001, ****P < 0.0001. Each experiment was repeated three times and results are means ± SD.

It has been established that extracellular HSP70 can be released from cells *via* several processes and considered as molecules with immunomodulatory functions (21). After overexpression of RP11-115N4.1, the HSP70 level in the supernatant of K562 cells increased significantly (**Figure 5A**). The serum of URSA samples with high RP11-115N4.1 expression was also tested for HSP70 level, and it was found to be significantly higher than the normal controls (**Figure 5B**). It was reported that exogenous HSP70 acts as a cytokine to human monocytes by stimulating a pro-inflammatory signal transduction cascade (22). In order to detect whether the increase of HSP70 affected monocytes after K562 cells overexpress RP11-115N4.1, the supernatant of K562 cells overexpressed RP11-115N4.1 was added to human monocytes and cultured for 12 hours. The pro-inflammatory factors including tumor necrosis factor (TNF)-α, interleukin (IL)-1β and interleukin (IL)-6 by ELISA in the serum increased significantly, that is, the level of pro-inflammatory factors in monocytes increased significantly (**Figure 5C**). In addition, the K562 supernatant overexpressing RP11-115N4.1 was incubated with trophoblast cell line Swan 71 for 24 h, and it was found that it inhibited Swan 71 cell migration (**Figure 5D**).

## Discussion

**Figure 6:**
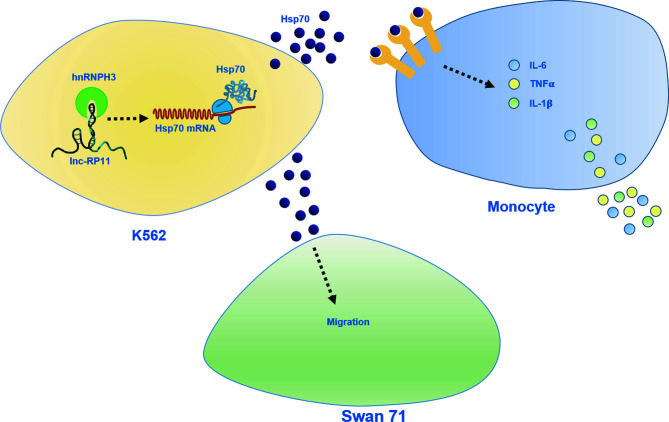
Schematic diagram of role of RP11-115N4.1. RP11-115N4.1 bound to HNRNPH3 and increased the protein level of HSP70 in K562 cells. HSP70 released outside the cell induced the upregulation of the inflammatory factor IL-6, IL-1*β* and TNF-*α* of monocytes and inhibited the migration of Swan 71. The black dashed line represents an unknown mechanism.

URSA is a common complication of pregnancy and the etiology is unknown (1–3). In our study, we sought to identify non-pregnant URSA-associated lncRNAs in PBMC. For the first time, we identified RP11-115N4.1 to be significantly upregulated in peripheral blood of URSA. Additionally, we explored the potential mechanisms by which RP11-115N4.1 binding to HNRNPH3 enhanced the expression level of HSP70, resulting in the release of inflammatory factors IL-6, IL-1β and TNF-*α* of monocytes, which might relate to the pathogenesis of URSA (**Figure 6**).

In our study, the heterogeneity of lncRNA expression profiles was observed in the URSA population. The increased expression of RP11-115N4.1 was observed in approximately 26.67% URSA patients (4/15), suggesting that the upregulated RP11-115N4.1 can explain the etiology for a small portion of URSA. Further, after analyzing clinical features, immune and biochemical changes in URSA patients with high and normal expression of RP11-115N4.1, we interestingly found that 3 out of 13 URSA patients with normal expression of RP11-115N4.1 had previous late miscarriage, while none was found in URSA patients with high expression of RP11-115N4.1. It is possible that the high expression of RP11-115N4.1 is more closely related to URSA with early abortion. To address this possibility, more samples are required to be tested. Till now, RP11-115N4.1 related research is rather less, and the mechanism of RP11-115N4.1 is poorly understood. RP11–115N4.1 was reported to be significantly downregulated in breast cancer tissues, and the RP11–115N4.1 overexpression inhibited mammary cell proliferation, migration *in vitro*, and repressed breast cancer proliferation and transference *in vivo* (23). Here, our findings demonstrated that RP11-115N4.1 overexpression significantly decreased the K562 cell proliferation, and modulated the immune response *via* activating HSP70 expression by binding to HNRNPH3. These findings strongly strengthen our understandings about molecular functions of RP11-115N4.1.

Previous studies reported that recurrent miscarriage was related to rheumatoid immune-related diseases, such as undifferentiated connective tissue diseases, antiphospholipid syndrome, antinuclear autoantibodies (24–26). Our previous study also found that mixed lymphocyte reaction-blocking factors is related to high expression of some rheumatoid biomarkers (27). In addition, Abdollahi E, et al. found that Interleukin-23 (IL-23) receptor and IL-23 signaling pathway may be related to RSA, rheumatoid arthritis and ankylosing spondylitis by affecting the growth and apoptosis of IL-17-producing T-helper-17 cells (28). In this study, a very strict inclusion criteria for RSA patients was applied, and RSA patients with known causes such as autoimmune disease and immunological factors were excluded. Therefore, the immune regulatory functions of RP11-115N4.1-HNRNPH3-HSP70 may be a patient-specific mechanism of URSA.

LncRNAs have been considered to perform multiple functions, such as regulating gene expression in cis or trans manners, organization of nuclear domains, and modulating the activity or abundance of proteins or RNAs (9, 29). LncRNAs play crucial roles in numerous cell functions, most of which need to interact with one or more RNA-binding proteins (30). On the one hand, most of them mainly regulate the expression of genes by connecting with transcription sites and interacting with proteins (31). On the other hand, some of them act as molecular decoys to prevent their association with DNA by binding to specific transcription factors (32). HNRNPH3 is one of the subfamily of heterogeneous nuclear ribonucleoproteins (HNRNPs) which are RNA binding proteins. HNRNPH3 mostly exists in the nucleus, but it is also expressed in the cytoplasm (33). This protein participates in early heat shock-induced splicing arrest by transiently leaving the HNRNP complexes (34). A study has found a novel HNRNPH3-ALK rearrangement in salivary duct carcinoma (35). RP11-115N4.1 transcript was mainly expressed in the cytoplasm but not in the nucleus (23). Therefore, we speculate that RP11-115N4.1 may bind to HNRNPH3 to rearrange the HNRNPH3 protein and affect some of its functions, leading to an increase in HSP70. On the other hand, the PPI map showed that HNRNPH3 interacted with SNRNP70, and in K562 cells, after overexpression of RP11-115N4.1, the expression of SNRNP70 mRNA increased (**Supplementary Figure 4**). Therefore, we speculate that RP11-115N4.1 affects the expression of SNRNP70 by binding to HNRNPH3, thereby increasing the expression of HSP70. However, the specific mechanism of upregulation of HSP70 expression by combining with HNRNPH3 remains to be revealed.

Heat-shock proteins (HSPs) function as a molecular chaperone, preserve cells during environmental, pathological or physiological stresses, as has been widely recognized (22, 36). Once leaked out of stressed cells, HSP70, by itself or binding with peptides, become a strong immunogen which can bind to pattern recognition receptors mainly expressed on antigen-presenting cells. High levels of HSP70 in peripheral blood maybe promote Th1 immune response and then lead to abortion (37). Our results observed that the pro-inflammatory factors including TNF-α, IL-1β and IL-6 in the serum of human monocytes increased significantly after culturing with the supernatant of K562 cells overexpressed RP11-115N4.1 for 12 hours. Previous studies reported that HSP70 can promote the production of IL-6, IL-1β and TNF-α in human monocytes by binding with high affinity to the plasma membrane and then stimulating a pro-inflammatory signal transduction cascade which dependent on intracellular calcium flux and nuclear factor (NF)-κB activation (22, 38). Our observations are generally consistent with these previous studies.

Whether these effects will enter the placenta through the circulatory system and affect the fetus in pregnancy? We found that the supernatants of K562 cell with RP11–115N4.1 overexpression significantly inhibited trophoblast cell migration *in vitro*, indicating that HSP70 or the induced pro-inflammatory cytokines are harmful to the trophoblast cells. Mei Han, et al. (39) found that trophoblast-derived peptides bound with HSP70 in-vitro, then activated T cells both *in vitro* and *in vivo* which promote Th1 immune response and specifically cause apoptosis of trophoblast, finally leading to abortion. Such activated T cells might influence fetal development in early pregnancy, rather than affecting preimplantation in a mouse model and suggest that complexes of trophoblastic peptides and HSP70 could be used as a novel contraceptive vaccine. The novel signaling, RP11-115N4.1- HNRNPH3-HSP70, may be the molecular mechanism of early abnormality in URSA patients, which still needs further study.

Although the functional effects of RP11-115N4.1-HNRNPH3-HSP70 signaling in URSA were investigated *in vitro*, our study still had some shortcomings. First, owing to our strict inclusion criteria, the sample size was relatively small. Large sample size will be further applied to strengthen our conclusions. Secondly, the detailed mechanism of HSP70 elevation induced by RP11-115N4.1 and HNRNPH3 interaction needs to be studied and explained. Thirdly, *in vivo* study using animal model is required to fully describe the causal relationship between RP11-115N4.1 and URSA. Fourthly, overexpression of RP11-115N4.1 in monocytes and its effects on lymphocytes were also required further investigation.

In conclusion, our results showed that RP11-115N4.1 bound to HNRNPH3 and thus enhanced the expression level of HSP70. Activated HSP70 functions as a cytokine, can enhance the production of IL-1β, IL-6 and TNF-α in human monocytes by stimulating inflammatory responses, which might relate to URSA. This novel signaling, RP11-115N4.1-HNRNPH3-HSP70, may be expected to be a novel etiological biomarker and a new therapeutic target for URSA.

## Data Availability Statement

The microarray data have been submitted to NCBI’s Gene Expression Omnibus (No. GSE179996). The RNA-seq raw sequence data have been deposited in the Genome Sequence Archive in National Genomics Data Center, China National Center for Bioinformation / Beijing Institute of Genomics, Chinese Academy of Science (No. HRA001020) (40, 41).

## Ethics Statement

The studies involving human participants were reviewed and approved by The Ethics Committee of Sun Yat-sen Memorial Hospital at Sun Yat-Sen University. The patients/participants provided their written informed consent to participate in this study. Written informed consent was obtained from the individual(s) for the publication of any potentially identifiable images or data included in this article.

## Author Contributions

These authors declare that they have contributed to this work and all meet the requirements of the authorship. HC designed the study and wrote the protocol. ML, XS, TD, DW, and YL performed research. LZ contributed important reagents. MZ participated in the production of pictures. KD, XN, and HM managed the literature searches and analyses. BH, LH, and LL were responsible for clinical specimen collection. JY and JZ undertook the statistical analysis. ML, XS, and CC wrote and revised the manuscript. All authors contributed to the article and approved the submitted version.

## Funding

This study was supported by the National Key Research and Development Program of China (2019YFA0801403), National Nature Science Foundation of China (81771660, 81741017 and 81770789), the Science and Technology Planning Project of Guangdong Province (2017A020214018 and 2017A020214003), Guangdong Natural Science Foundation (2018A030313023, 2018A030313162, 2018A030310162, and 18zxxt56), the Science and Technology Planning Project of Guangzhou City Central Universities (201704020034), 5010 projects at Sun Yat-Sen University (2012006), Science and Technology Planning Project of Guangdong Province (pdjh2020b0010), Xinjiang Uyghur Autonomous Region Project of Science and Technology (2020E02129).

## Conflict of Interest

The authors declare that the research was conducted in the absence of any commercial or financial relationships that could be construed as a potential conflict of interest.

## Publisher’s Note

All claims expressed in this article are solely those of the authors and do not necessarily represent those of their affiliated organizations, or those of the publisher, the editors and the reviewers. Any product that may be evaluated in this article, or claim that may be made by its manufacturer, is not guaranteed or endorsed by the publisher.

## References

[1] RCOG. Royal College of Obstetricians and Gynaecologists Recurrent Miscarriage, Investigation and Treatment of Couples (Green-Top 17) (2011). Available at: https://www.rcog.org.uk/en/guidelines-research-services/guidelines/gtg17/ (Accessed May15, 2021).

[2] Cozzolino M Rizzello F Riviello C Romanelli C Coccia Elisabetta M. Ongoing Pregnancies in Patients With Unexplained Recurrent Pregnancy Loss: Adverse Obstetric Outcomes. Hum Fertil (Camb) (2019) 22(3):219–25. 10.1080/14647273.2018.1475754 29793356

[3] Ali S Majid S Niamat Ali M Taing S El-Serehy HA Al-Misned FA. Evaluation of Etiology and Pregnancy Outcome in Recurrent Miscarriage Patients. Saudi J Biol Sci (2020) 27(10):2809–17. 10.1016/j.sjbs.2020.06.049 PMC7499272 32994741

[4] Popescu F Jaslow CR Kutteh WH. Recurrent Pregnancy Loss Evaluation Combined With 24-Chromosome Microarray of Miscarriage Tissue Provides a Probable or Definite Cause of Pregnancy Loss in Over 90% of Patients. Hum Reprod (2018) 33(4):579–87. 10.1093/humrep/dey021 29538673

[5] Muyayalo KP Li ZH Mor G Liao AH. Modulatory Effect of Intravenous Immunoglobulin on Th17/Treg Cell Balance in Women With Unexplained Recurrent Spontaneous Abortion. Am J Reprod Immunol (2018) 80(4):e13018. 10.1111/aji.13018 29984444

[6] Motedayyen H Rezaei A Zarnani AH Tajik N. Human Amniotic Epithelial Cells Inhibit Activation and Pro-Inflammatory Cytokines Production of Naive CD4+ T Cells From Women With Unexplained Recurrent Spontaneous Abortion. ReprodBiol (2018) 18(2):182–8. 10.1016/j.repbio.2018.04.002 29729842

[7] Deshmukh H Way SS. Immunological Basis for Recurrent Fetal Loss and Pregnancy Complications. Annu Rev Pathol (2019) 14:185–210. 10.1146/annurev-pathmechdis-012418-012743 30183507 PMC6566855

[8] Abdolmohammadi Vahid S Ghaebi M Ahmadi M Nouri M Danaei S Aghebati-Maleki L. Altered T-Cell Subpopulations in Recurrent Pregnancy Loss Patients With Cellular Immune Abnormalities. J Cell Physiol (2019) 234(4):4924–33. 10.1002/jcp.27290 30187472

[9] Kopp F Mendell JT. Functional Classification and Experimental Dissection of Long Noncoding RNAs. Cell (2018) 172(3):393–407. 10.1016/j.cell.2018.01.011 29373828 PMC5978744

[10] Uszczynska-Ratajczak B Lagarde J Frankish A Guigó R Johnson R. Towards a Complete Map of the Human Long Non-Coding RNA Transcriptome. Nat Rev Genet (2018) 19(9):535–48. 10.1038/s41576-018-0017-y PMC6451964 29795125

[11] Allou L Balzano S Magg A Quinodoz M Royer-Bertrand B Schöpflin R. Non-Coding Deletions Identify Maenli lncRNA as a Limb-Specific En1 Regulator. Nature (2021) 592(7852):93–8. 10.1038/s41586-021-03208-9 33568816

[12] Huang W Zhou H Pi L Xu Y Fu L Yang Y. Association Between the Rs2288947 Polymorphism of the lncRNA TINCR Gene and the Risk of Recurrent Miscarriage in a Southern Chinese Population. J Clin Lab Anal (2019) 33(6):e22919. 10.1002/jcla.22919 31124188 PMC6642304

[13] Che D Huang W Fang Z Li L Wu H Pi L. The lncRNA CCAT2 Rs6983267 G Allele Is Associated With Decreased Susceptibility to Recurrent Miscarriage. J Cell Physiol (2019) 234(11):20577–83. 10.1002/jcp.28661 30982978

[14] Huang Z Du G Huang X Han L Han X Xu B. The Enhancer RNA lnc-SLC4A1-1 Epigenetically Regulates Unexplained Recurrent Pregnancy Loss (URPL) by Activating CXCL8 and NF-kB Pathway. EBioMedicine (2018) 38:162–70. 10.1016/j.ebiom.2018.11.015 PMC6306333 30448228

[15] Zhu L Liu M Zhang S Ou Y Chen Y Wei J. Foxp3 TSDR Hypermethylation Is Correlated With Decreased Tregs in Patients With Unexplained Recurrent Spontaneous Abortion. Reprod Sci (2021) 28(2):470–8. 10.1007/s43032-020-00299-z 32839941

[16] Bolger AM Lohse M Usadel B. Trimmomatic: A Flexible Trimmer for Illumina Sequence Data. Bioinformatics (2014) 30(15):2114–20. 10.1093/bioinformatics/btu170 PMC4103590 24695404

[17] Trapnell C Pachter L Salzberg SL. TopHat: Discovering Splice Junctions With RNA-Seq. Bioinformatics (2009) 25(9):1105–11. 10.1093/bioinformatics/btp120 PMC2672628 19289445

[18] Robinson MD McCarthy DJ Smyth GK. Edger: A Bioconductor Package for Differential Expression Analysis of Digital Gene Expression Data. Bioinformatics (2010) 26(1):139–40. 10.1093/bioinformatics/btp616 PMC2796818 19910308

[19] Ge SX Jung D Yao R. ShinyGO: A Graphical Gene-Set Enrichment Tool for Animals and Plants. Bioinformatics (2020) 36(8):2628–9. 10.1093/bioinformatics/btz931 PMC7178415 31882993

[20] Wang KC Chang HY. Molecular Mechanisms of Long Noncoding RNAs. Mol Cell (2011) 43(6):904–14. 10.1016/j.molcel.2011.08.018 PMC3199020 21925379

[21] Radons J. The Human HSP70 Family of Chaperones: Where do We Stand? Cell Stress Chaperones (2016) 21(3):379–404. 10.1007/s12192-016-0676-6 26865365 PMC4837186

[22] Asea A Kraeft SK Kurt-Jones EA Stevenson MA Chen LB Finberg RW. HSP70 Stimulates Cytokine Production Through a CD14-Dependant Pathway, Demonstrating Its Dual Role as a Chaperone and Cytokine. Nat Med (2000) 6(4):435–42. 10.1038/74697 10742151

[23] Jiang Y Lin L Zhong S Cai Y Zhang F Wang X. Overexpression of Novel lncRNA NLIPMT Inhibits Metastasis by Reducing Phosphorylated Glycogen Synthase Kinase 3β in Breast Cancer. J Cell Physiol (2019) 234(7):10698–708. 10.1002/jcp.27738 30417392

[24] Yang S Ni R Lu Y Wang S Xie F Zhang C. A Three-Arm, Multicenter, Open-Label Randomized Controlled Trial of Hydroxychloroquine and Low-Dose Prednisone to Treat Recurrent Pregnancy Loss in Women With Undifferentiated Connective Tissue Diseases: Protocol for the Immunosuppressant Regimens for LIving FEtuses (ILIFE) Trial. Trials (2020) 21(1):771. 10.1186/s13063-020-04716-1 32907619 PMC7488113

[25] Santos TDS Ieque AL de Carvalho HC Sell AM Lonardoni MVC Demarchi IG. Antiphospholipid Syndrome and Recurrent Miscarriage: A Systematic Review and Meta-Analysis. J Reprod Immunol (2017) 123:78–87. 10.1016/j.jri.2017.09.007 28985591

[26] Ticconi C Pietropolli A Borelli B Bruno V Piccione E Bernardini S. Antinuclear Autoantibodies and Pregnancy Outcome in Women With Unexplained Recurrent Miscarriage. Am J Reprod Immunol (2016) 76(5):396–9. 10.1111/aji.12560 27616598

[27] Meng L Tan J Du T Lin X Zhang S Nie X. The Effects of LIT and MLR-Bf on Immune Biomarkers and Pregnancy Outcomes in Women With Previous Early Recurrent Miscarriage: A Retrospective Study. Front Immunol (2021) 12:642120. 10.3389/fimmu.2021.642120 34017330 PMC8129162

[28] Abdollahi E Tavasolian F Momtazi-Borojeni AA Samadi M Rafatpanah H. Protective Role of R381Q (Rs11209026) Polymorphism in IL-23R Gene in Immune-Mediated Diseases: A Comprehensive Review. J Immunotoxicol (2016) 13(3):286–300. 10.3109/1547691X.2015.1115448 27043356

[29] Ulitsky I Bartel DP. lincRNAs: Genomics, Evolution, and Mechanisms. Cell (2013) 154(1):26–46. 10.1016/j.cell.2013.06.020 23827673 PMC3924787

[30] Zhu J Fu H Wu Y Zheng X. Function of lncRNAs and Approaches to lncRNA-Protein Interactions. Sci China Life Sci (2013) 56(10):876–85. 10.1007/s11427-013-4553-6 24091684

[31] Wang X Arai S Song X Reichart D Du K Pascual G. Induced ncRNAs Allosterically Modify RNA-Binding Proteins in Cis to Inhibit Transcription. Nature (2008) 454(7200):126–30. 10.1038/nature06992 PMC2823488 18509338

[32] Geisler S Coller J. RNA in Unexpected Places: Long non-Coding RNA Functions in Diverse Cellular Contexts. Nat Rev Mol Cell Biol (2013) 14(11):699–712. 10.1038/nrm3679 24105322 PMC4852478

[33] Cho KI Yoon D Qiu S Danziger Z Grill WM Wetsel WC. Loss of Ranbp2 in Motoneurons Causes Disruption of Nucleocytoplasmic and Chemokine Signaling, Proteostasis of Hnrnph3 and Mmp28, and Development of Amyotrophic Lateral Sclerosis-Like Syndromes. Dis Model Mech (2017) 10(5):559–79. 10.1242/dmm.027730 PMC5451164 28100513

[34] Gattoni R Mahé D Mähl P Fischer N Mattei MG Stévenin J. The Human hnRNP-M Proteins: Structure and Relation With Early Heat Shock-Induced Splicing Arrest and Chromosome Mapping. Nucleic Acids Res (1996) 24(13):2535–42. 10.1093/nar/24.13.2535 PMC145970 8692693

[35] Dogan S Ng CKY Xu B Kumar R Wang L Edelweiss M. The Repertoire of Genetic Alterations in Salivary Duct Carcinoma Including a Novel HNRNPH3-ALK Rearrangement. Hum Pathol (2019) 88:66–77. 10.1016/j.humpath.2019.03.004 30946933 PMC7388159

[36] Craig EA Gross CA. Is HSP70 the Cellular Thermometer? Trends Biochem Sci (1991) 16(4):135–40. 10.1016/0968-0004(91)90055-z 1877088

[37] De Maio A Vazquez D. Extracellular Heat Shock Proteins: A New Location, a New Function. Shock (2013) 40(4):239–46. 10.1097/SHK.0b013e3182a185ab PMC4351735 23807250

[38] Zhang FX Kirschning CJ Mancinelli R Xu XP Jin Y Faure E. Bacterial Lipopolysaccharide Activates Nuclear Factor-kappaB Through Interleukin-1 Signaling Mediators in Cultured Human Dermal Endothelial Cells and Mononuclear Phagocytes. J Biol Chem (1999) 274(12):7611–4. 10.1074/jbc.274.12.7611 10075645

[39] Gulic T Laskarin G Dominovic M GlavanGacanin L Babarovic E Haller H. Potential Role of Heat-Shock Protein 70 and Interleukin-15 in the Pathogenesis of Threatened Spontaneous Abortions. Am J Reprod Immunol (2016) 76(2):126–36. 10.1111/aji.12525 27225940

[40] Wang Y Song F Zhu J Zhang S Yang Y Chen T. GSA: Genome Sequence Archive. Genomics Proteomics Bioinf (2017) 15(1):14–8. 10.1016/j.gpb.2017.01.001 PMC5339404 28387199

[41] CNCB-NGDC Members and Partners. Database Resources of the National Genomics Data Center, China National Center for Bioinformation in 2021. Nucleic Acids Res (2021) 49(D1):D18–28. 10.1093/nar/gkaa1022 PMC7779035 33175170

