# Supplementary material for: Long Noncoding RNA RP11-115N4.1 Promotes Inflammatory Responses by Interacting With HNRNPH3 and Enhancing the Transcription of HSP70 in Unexplained Recurrent Spontaneous Abortion
Source: Front Immunol. 2021 Aug 13;12:717785. doi: 10.3389/fimmu.2021.717785 (PMC8414257; doi:10.3389/fimmu.2021.717785)
Supplement: Supplementary file 1 [file DataSheet_1.pdf]

Supplementary Table 1. Abbreviations and reference values of immune and biochemical markers

Variables	Immune and biochemical markers	Normal reference values
**MLR-Bf**	Mixed lymphocyte reaction-blocking factors	positive
**White blood cells (10^9/L)**	~	3.50-9.50
**Lymphocytes (10^9/L)**	~	1.10-3.20
**Anti-β2GP1-Ab -IgG (U/mL)**	Anti-β2-glycoprotein 1 antibody-immunoglobulin G	<20: negative; 20-39: low titer positive; ≥40: high titer positive
**Anti-β2GP1-Ab -IgM (U/mL)**	Anti-β2-glycoprotein 1 antibody-immunoglobulin M	<20: negative; 20-39: low titer positive; ≥40: high titer positive
**Anti-β2GP1-Ab -IgA (U/mL)**	Anti-β2-glycoprotein 1 antibody-immunoglobulin A	<20: negative; 20-39: low titer positive; ≥40: high titer positive
**ACA-IgG (U/mL)**	Anti-cardiolipin antibody-immunoglobulin G	<20: negative; 20-39: low titer positive; ≥40: high titer positive
**ACA-IgM (U/mL)**	Anti-cardiolipin antibody-immunoglobulin M	<20: negative; 20-39: low titer positive; ≥40: high titer positive
**ACA-IgA (U/mL)**	Anti-cardiolipin antibody-immunoglobulin A	<20: negative; 20-39: low titer positive; ≥40: high titer positive
**Hcy (μmol/L)**	Homocysteine	0.0-15.0
**ADP (%)**	Platelet aggregation function (adenosine diphosphate)	37.3-47.5
**aPS/PT-IgG (U/mL)**	Anti-Phosphatidylserine/Prothrombin antibody - immunoglobulin G	<30: negative; ≥30: positive
**aPS/PT-IgM (U/mL)**	Anti-Phosphatidylserine/Prothrombin antibody - immunoglobulin M	<30: negative; ≥30: positive

Supplementary Table 2. Primers for real time qPCR

Gene		Sequences of primer (5'-3')
GAPDH	forward	CATGAGAAGTATGACAACAGCCT
	reward	AGTCCTTCCACGATACCAAAGT
RP11-115N4.1	forward	GCCCACCACAACCATTCTT
	reward	CAATGGCTGGAGGAAGAGGA
TRIM47	forward	CTGAGCAGTCCAAAGTCCTGA
	reward	CTACGGCTGCACTCTTGATG
SCN4A	forward	CATCGTACTCAACAAGGGCAA
	reward	CGCCTGACTACGCTGAAGG
CHAC1	forward	GAACCCTGGTTACCTGGGC
	reward	CGCAGCAAGTATTCAAGGTTGT
CHMP1A	forward	GTGTATGCCGAGAACGCCAT
	reward	TTGGAGGCCACTGCGTCTA
NACC1	forward	ACCCGAGTAAAGCATGAAGCC
	reward	GACACGGGGCAGTTTGAGAG
XBP1	forward	CCCTCCAGAACATCTCCCCAT
	reward	ACATGACTGGGTCCAAGTTGT
INHBE	forward	ATCTTCCGATGGGGACCAAG
	reward	AGAGTTAAGGTATGCCAGCCC
GABRE	forward	AAGCCTCTTCCCGTGATGTTG
	reward	CTGCTCCCAGTCTCAGTCTCA
HNRNPH3	forward	AATGGTCCAAATGACGCTAGTG
	reward	CTCCCCTGGTAGTCCATCGT
HSPA8	forward	ACCTACTCTTGTGTGGGTGTT
	reward	GACATAGCTTGGAGTGGTTCG
HSPA1A	forward	TAACCCCATCATCAGCGGAC
	reward	AACAGCAATCTTGGAAAGGCCC
HSPA1B	forward	TCAGGCCCTACCATTGAGGAG
	reward	CTTGAGTCCCAACAGTCCACC

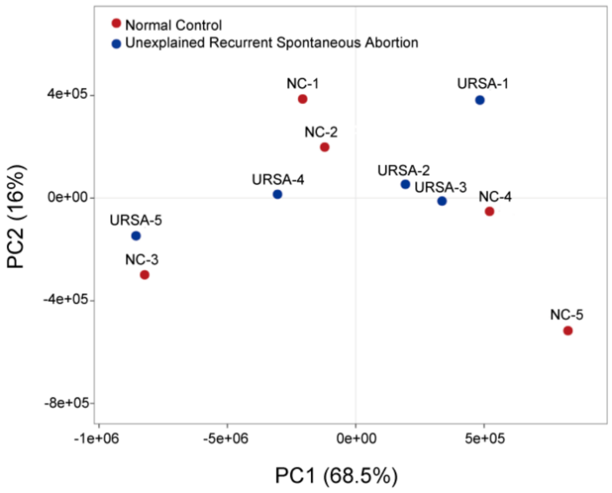

Supplementary Figure 1. Cluster map of URSA samples and normal controls (5 *vs.* 5).

Supplementary Table3. Comparison of clinical features, levels of immune and biochemical markers between group 1 and group 2 (variables are presented as mean ± SD or number/percentage or medians with lowest and highest values)

Variables	Group（*N*=7）	Group2（*N*=13）	*Z*	*P*-value
**Age (year)**	30.14 ± 3.98	31.69 ± 4.53	~	0.458
**Height (cm)**	156.43 ± 3.55	159.35 ± 5.72	~	0.238
**BMI (kg/m^2)**	21.29 ± 2.74	20.97 ± 3.15	~	0.820
**local**				
Urban	5 (71.43%)	11 (84.62%)	~	0.587
Rural	2 (28.57%)	2 (15.38%)		
**Regular menstruation period**				
Yes	5 (71.43%)	10 (76.92%)	~	1.00
No	2 (28.57%)	3 (23.08%)		
**Previous assistant reproduction**				
Yes	1 (14.29%)	1 (7.69%)	~	1.00
No	6 (85.71%)	12 (92.31%)		
**Number of gravidities**	3.71 ± 0.95	3.69 ± 0.75	~	0.955
**Number of live births**	0 (0, 0)	0 (0, 1)	-1.066	0.286
**Number of spontaneous abortions**	3.43 ± 0.79	3.23 ± 0.44	~	0.475
**Previous early miscarriage**				
2	0 (0.00%)	2 (15.38%)	~	0.521
≥3	7 (100%)	11 (84.62%)		
**Previous late miscarriage**				
**0**	**0 (0.00%)**	**3 (23.08%)**	~	**0.521**
**≥1**	**7 (100%)**	**10 (76.92%)**		
**Previous preterm birth**				
0	0 (0.00%)	0 (0.00%)	~	1.000

≥1	7 (100%)	13 (100 %)		
**MLR-Bf (+):**	0.00% (0/7)	15.38% (2/13)	~	0.521
**White blood cells (10^9/L)**	5.23 ± 0.80	5.78 ± 1.45		0.364
**Lymphocytes (10^9/L)**	1.83 ± 0.52	1.95 ± 0.59	~	0.650
**T cells (CD3+, %)**	68.47 ± 6.82	71.42 ± 5.67	~	0.315
**B cells (CD3−CD19+, %)**	12.36 ± 3.48	11.41 ± 3.59	~	0.574
**NK cells (CD3−CD16+CD56+, %)**	18.40 ± 6.47	16.73 ± 4.62	~	0.512
**CD4+T cells/lymphocytes (CD3+CD4+, %)**	37.05 ± 4.65	36.31 ± 5.32	~	0.762
**CD8+T cells/lymphocytes (CD3+CD8+, %)**	25.68 ± 8.17	26.64 ± 5.48	~	0.757
**CD3+CD4+/CD3+CD8+**	1.62 ± 0.70	1.43 ± 0.43	~	0.462
**CIK cells (CD3+CD56+, %)**	2.23 ± 2.40	2.20 ± 1.66	~	0.978
**Anti-βGP1-IgG**[a]	0.00% (0/7)	0.00% (0/13)	~	1.000
**Anti-βGP1-IgM**[a]	0.00% (0/7)	0.00% (0/13)	~	1.000
**Anti-βGP1-IgA**[a]	0.00% (0/7)	0.00% (0/13)	~	1.000
**ACA-IgG**[a]	0.00% (0/7)	0.00% (0/13)	~	1.000
**ACA-IgM**[a]	0.00% (0/7)	0.00% (0/13)	~	1.000
**ACA-IgA**[a]	0.00% (0/7)	0.00% (0/13)	~	1.000
**Hcy (µmol/L)**	9.12 ± 2.04	8.58 ± 2.00	~	0.575
**ADP (%)**	43.13 ± 3.46	42.15 ± 5.88	**~**	0.694
**aPS/PT-IgG**[a]	0.00% (0/7)	0.00% (0/13)	~	1.000
**aPS/PT-IgM**[a]	0.00% (0/7)	0.00% (0/13)	~	1.000

Group 1: high expression of RP11-115N4.1; Group 2: normal expression of RP11-115N4.1; [a]: The percentage of positive cases

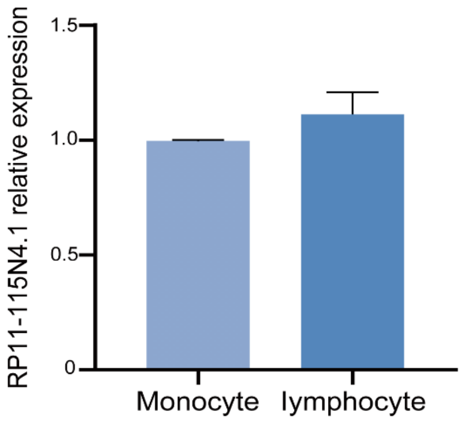

Supplementary Figure 2. Relative expression of RP11-115N4.1 in monocytes and lymphocytes. *GAPDH* was used as an internal control. Each experiment was repeated three times and results are means ± SD.

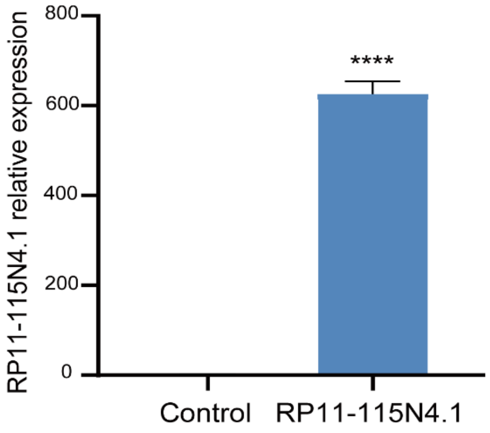

Supplementary Figure 3. Relative expression of the overexpressed RP11-115N4.1 after transfection in K562 cells. *GAPDH* was used as an internal control. **P*< 0.05, ***P*< 0.01, ****P*< 0.001, *****P*< 0.0001. Each experiment was repeated three times and results are means ± SD.

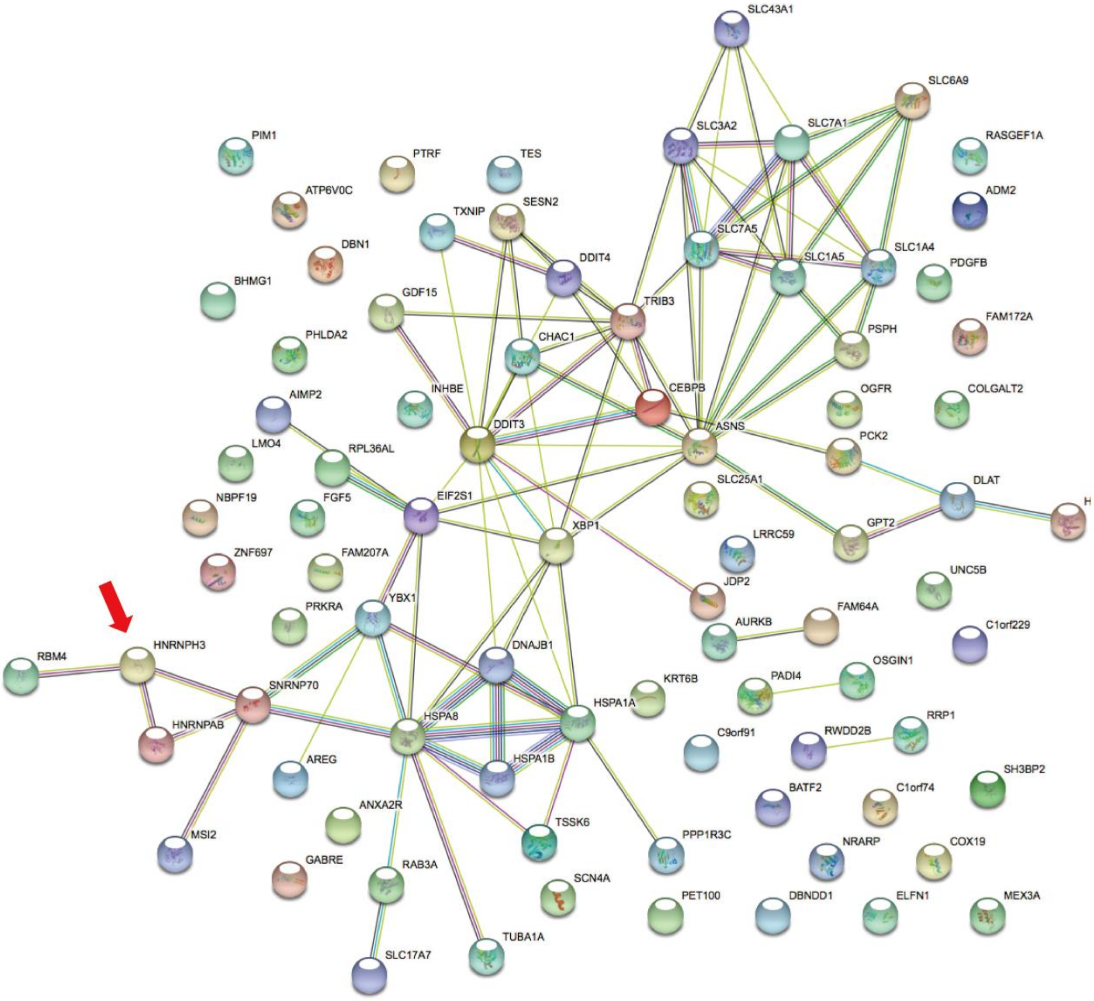

Supplementary Figure 4. The protein-protein interaction map of the differential genes of K562 cells after overexpression of RP11-115N4.1 ($P< 0.001$) and RP11-115N4.1 pull down mass spectrometry differential genes. (**Up-regulated genes:** *HSPA1A*, *LINC00641*, *HSPA1B*, *RPL36AL*, *GABRE*, *PHLDA2*, *BAIAP2-DT*, *RASGEF1A*, *SLC17A7*, *RAB3A*, *MEX3A*, *DNAJB1*, *ATP6V0C*, *CAVIN1*, *FAM207A*, *HSPA8*, *NRARP*, *OGFR*, *OSGIN1*, *SNRNP70*, *DBNDD1*, *HMGCS1*, *NBPF14*, *PDGFB*, *PET100*, *PIMREG*, *DBN1*, *FGF5*, *RRP1*, *FAM172A*, *AURKB*. **Down-regulated gene**：*TRIB3*, *DDIT4*, *SESN2*, *INHBE*, *ANXA2R*, *PSPH*, *SH3BP2*, *JDP2*, *H1-4*, *SLC3A2*, *PPP1R3C*, *ASNS*, *DDIT3*, *PCK2*, *SLC7A1*, *CEBPB*, *GDF15*, *SNHG1*, *SLC1A4*, *TXNIP*, *ELFN1*, *AREG*, *UNC5B*, *SLC1A5*, *PIM1*, *SLC43A1*, *CHAC1*, *C1orf229*, *SLC7A5*, *SLC6A9*, *ZNF697*, *XBP1*, *BATF2*, *RWDD2B*, *ADM2*, *TES*, *COX19*, *CYP2T1P*, *BHMG1*, *TSSK6*, *SCN4A*, *TMEM268*, *COLGALT2*, *GPT2*, *C1orf74*, *LMO4*, *BHMG1*, *TSSK6*, *SCN4A*, *TMEM268*, *COLGALT2*, *GPT2*, *C1orf74*, *LMO4*. **Mass spectrometry differential genes:** *HNRNPH3*, *RBM4*, *TUBA1A*, *KRT6B*, *DLAT*).

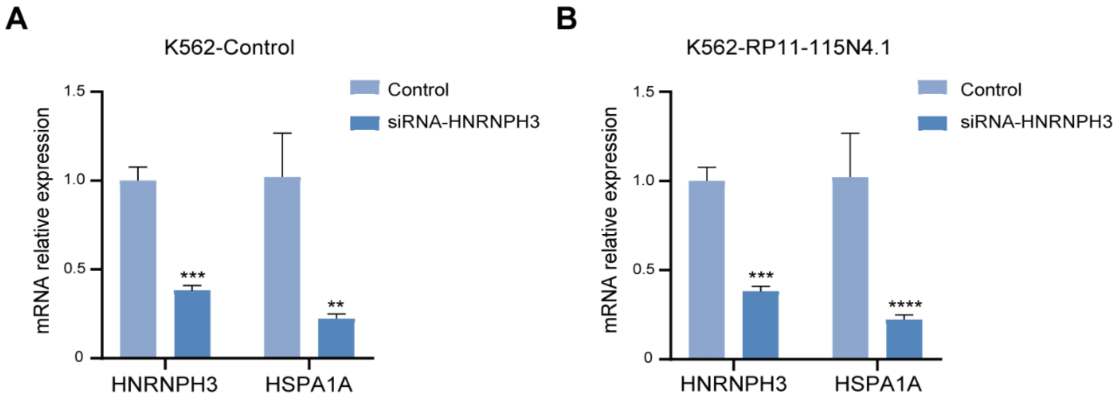

Supplementary Figure 5. (A, B) qPCR detection of changes in HSPA1A mRNA levels after knocking down HNRNPH3. *GAPDH* was used as an internal control. **P*< 0.05, ***P*< 0.01, ****P*< 0.001, *****P*< 0.0001. Each experiment was repeated three times and results are means ± SD.
